# Supplementary material for: The prevalence, spatial distribution and geographic weighted regression of open defecation practice in sub-Saharan Africa using demographic and health survey (DHS) data
Source: PLoS One. 2025 Dec 17;20(12):e0336938. doi: 10.1371/journal.pone.0336938 (PMC12711022; doi:10.1371/journal.pone.0336938)
Supplement: S1 File — (DOCX) [file pone.0336938.s001.docx]

| Country | DHS year | Sample Size | Open defecation practice in percent |
| --- | --- | --- | --- |
| Angola | 2015-16 | 16,109 | 35.23 |
| Benin | 2017-18 | 14,156 | 54.08 |
| Burkina Faso | 2021 | 13,251 | 32.29 |
| Burundi | 2016-17 | 15,977 | 2.58 |
| Cameroon | 2018 | 11,710 | 4.32 |
| Chad | 2014–2015 | 17,233 | 71.22 |
| Comoros | 2012 | 4,482 | 0.89 |
| Congo Democratic Republic | 2013-14 | 18,171 | 16.90 |
| Cote d'Ivoire | 2021 | 14,766 | 22.08 |
| Ethiopia | 2016 | 16,650 | 35.22 |
| Gabon | 2019-21 | 11,781 | 1.40 |
| Gambia | 2019-20 | 6,549 | 1.48 |
| Ghana | 2022 | 17,933 | 28.70 |
| Guinea | 2018 | 7,912 | 14.27 |
| Kenya | 2022 | 37,911 | 10.15 |
| Lesotho | 2023-24 | 9,810 | 22.66 |
| Liberia | 2019-20 | 9,068 | 45.36 |
| Madagascar | 2021 | 20,510 | 36.50 |
| Malawi | 2015-16 | 26,361 | 5.93 |
| Mali | 2018 | 9,510 | 14.19 |
| Mauritania | 2019-21 | 11,658 | 33.80 |
| Mozambique | 2022-23 | 14,250 | 23.06 |
| Namibia | 2013 | 9,849 | 47.45 |
| Niger | 2012 | 10,750 | 64.42 |
| Nigeria | 2018 | 40,427 | 28.11 |
| Rwanda | 2019-20 | 12,949 | 3.09 |
| Senegal | 2023 | 8,423 | 10.46 |
| Sierra Leone | 2019 | 13,399 | 20.40 |
| South Africa | 2016 | 11,083 | 3.05 |
| Tanzania | 2022 | 15,705 | 8.98 |
| Togo | 2022 | 9,549 | 54.10 |
| Uganda | 2016 | 19,588 | 9.19 |
| Zambia | 2018 | 12,831 | 10.65 |
| Zimbabwe | 2015 | 10,534 | 20.68 |
