# Supplementary material for: The prevalence, spatial distribution and geographic weighted regression of open defecation practice in sub-Saharan Africa using demographic and health survey (DHS) data
Source: PLoS One. 2025 Dec 17;20(12):e0336938. doi: 10.1371/journal.pone.0336938 (PMC12711022; doi:10.1371/journal.pone.0336938)
Supplement: Attachment — Submitted filename: PLOS ONE Review.pdf [file pone.0336938.s002.pdf]

PLOS ONE Review

Title:- The Prevalence, Spatial Distribution and Geographic Weighted Regression of Open Defecation Practice in sub-Saharan Africa Using Demographic and Health Survey (DHS) Data
Authors:- Lidetu Demoze et al

Summary

This study analyzes open defecation prevalence and spatial patterns across 34 sub-Saharan African countries using DHS data. The authors employ spatial statistics (Moran's I, Getis-Ord Gi, SaTScan) and Geographically Weighted Regression (GWR) to identify hotspots and associated factors. Findings indicate a 23.24 percent prevalence with significant geographic clustering and links to education, water access, and poverty

Comments to the Author

- ⇨ Technical Soundness & Data Support for Conclusions

The study is technically sound, with data largely supporting conclusions. However, clarify causality limitations due to cross-sectional design and address counterintuitive findings (e.g., negative association of urban/rural residency with open defecation)

- ⇨ Statistical Rigor
  - ✓ Assessment:-
    - Generally acceptable with minor clarifications needed
  - ✓ Spatial analyses are robust, but provide:-
    - Timeframe of DHS data per country
    - Multicollinearity assessment in GWR
    - Criteria for cluster/household inclusion.
- ⇨ Methodological Standards
  - ✓ Adheres to spatial epidemiology standards but requires:-
    - Enhanced documentation of variable selection for GWR
    - Justification for bandwidth selection in spatial analyses.
- ⇨ Conclusion Support
  - ✓ Conclusions align with results, but strengthen by:-
    - Discussing drivers behind spatial clusters
    - Integrating policy implications for identified hotspots.
- ⇨ Data Repository Compliance
  - ✓ Data availability statement is appropriate, citing DHS program access. Ensure supplementary country-level statistics are included.
- ⇨ Presentation & Language
  - ✓ Generally intelligible but requires:-
    - Minor English editing for conciseness
    - Higher-resolution maps with clear legends/scales.
- ⇨ Detailed Feedback
  - ✓ Strengths
    - Addresses critical public health challenge aligned with SDG 6
    - Rigorous spatial methodology with large representative sample
    - Clear structure and contextualization.
- ⇨ Major Issues
  - ✓ Methods Detail:-
    - Specify temporal range of DHS datasets used.

        - Detail handling of missing data and variable coding.
        - Please clarify the time frame of the DHS data included for each country. Specify inclusion/exclusion criteria for clusters and households, and provide more detail on how variables were selected and handled in the GWR, including how you addressed multicollinearity
    - ✓ Interpretation of Spatial Results and Conclusion:-
        - Assessment:-Valid but requires more cautious language

        - The conclusion that larger households are negatively associated with open defecation may seem counterintuitive and could benefit from referencing cultural/family dynamics or sanitation sharing behavior.
        - Statements like "richest households negatively associated" need caution:-correlation ≠ causation, and the pathways should be clarified.
        - Explain mechanisms driving East/West African hotspots beyond statistical identification.
        - The spatial analyses are robust, but the discussion would benefit from deeper interpretation of the drivers behind identified hotspots and coldspots, and more explicit policy implications for regions with high prevalence
- ⇨ Causal Inference and Confounding:-
    - ✓ Emphasize cross-sectional limitations regarding causal inference in discussion.
    - ✓ Discuss potential unmeasured confounders
- ⇨ Urban vs. Rural Residency:-
    - ✓ The finding that both urban and rural residency are negatively associated with open defecation is counterintuitive. Please clarify this result and discuss possible explanations or limitations in the data or analytic approach.
- ⇨ Minor Issues
    - ✓ Tables/Figures:-
        - Ensure that all figures/maps are high-resolution and include legends and scales for clarity.
    - ✓ Ethics and Data Statements:-
        - Assessment:-Fully compliant
        - The ethics and data availability statements are appropriate and transparent, but consider explicitly listing the countries and IRBs where possible IRBs.
        - Clearly stated that this is a secondary analysis of DHS data, which had ethical clearance and informed consent in all countries.
    - ✓ Language:-
        - The manuscript is generally clear but would benefit from minor English language editing for conciseness and flow.
- ⇨ Suggestions
    - ✓ Include a limitations subsection addressing:-
        - Regional data gaps (e.g., unstable regions not covered)
        - Temporal variations in DHS collection.
        - Expand policy recommendations targeting hotspot interventions.
- ⇨ Comments to the Authors
    - ✓ Strengths

- The study addresses a major public health and development challenge in sub-Saharan Africa, with direct relevance to SDG 6.
- Use of large representative DHS datasets and advanced spatial methods adds rigor and regional nuance.
- The Study is generally well-structured, with clear aims, methods, and results.

⇨ Comments to the Editor

✓ Competing Interests:-

- The authors declare no competing interests.

✓ Funding:-

- The authors received no specific funding for this work.

✓ Ethics:-

- This is a secondary analysis of publicly available, de identified data original surveys obtained ethical clearance from national review boards and informed consent from participants.

✓ Data Availability:-

- Data are available from the DHS program website.

⇨ Recommendation

✓ Major Revision

This Study addresses an urgent public health issue with advanced methodology and important findings. However, it would benefit from clarification of methods expanded interpretation of spatial results and minor language editing.

PLOS ONE Publication Review

⇨ Summary of PLOS ONE Criteria Assessment

Criterion	Status
Scientific validity	Acceptable
Ethical standards	Fully met
Data availability	Compliant
Statistical methods	Needs clarification
Writing quality	Needs editing
Relevance to scope of PLOS ONE	Within scope
Overall recommendation	Minor Revision
