# Supplementary material for: The prevalence, spatial distribution and geographic weighted regression of open defecation practice in sub-Saharan Africa using demographic and health survey (DHS) data
Source: PLoS One. 2025 Dec 17;20(12):e0336938. doi: 10.1371/journal.pone.0336938 (PMC12711022; doi:10.1371/journal.pone.0336938)
Supplement: Attachment — Submitted filename: Reviewers comment_Open Defecation.docx [file pone.0336938.s003.docx]

Reviewers comment

The article investigates “The Prevalence, Spatial Distribution, and Geographic Weighted Regression of Open Defecation Practices in sub-Saharan Africa Using Demographic and Health Survey (DHS) Data.” The authors establish a strong foundation for their study by clearly identifying research gaps and employing appropriate methodologies to address these deficiencies. The sample size is adequate, and the data analysis tools used are sufficient. The methodology of the study is robust, ensuring reproducibility. Overall, it offers valuable insights into open defecation in sub-Saharan Africa, providing evidence that could inform WASH interventions, policy, and practice. However, the authors do not emphasise the key novel contributions of the study. Although a wealth of results is presented, the discussions fail to thoroughly build on the results, explore the practical implications of these findings and how they can be applied to stimulate interventions to effectively reduce open defecation. I suggest authors improve the level of English used for the write-up.

Specific comments:

1. The section on abstract: The abstract gives a summary of the study, providing essential details such as the basis for the study, sample size, methodology, results, and discussions. The abstract can be strengthened by the following modifications.

- The conclusion is a repeat of the methodology and the last sentence of the background. The conclusion should highlight the key finding(s) and their implication on policy and future research. What makes this study novel?
- The study asserts 23.24% of open defecation practices in Sub-Saharan Africa. How does this compare to the value obtained by the JMP? This should be highlighted briefly.

2. The section on introduction: The introduction provides a good background to the study, citing relevant literature and establishing the basis for the study. However, the write-up is disjointed and lacks chronology. Literature on prevalence, access to WASH services, and global and regional contexts of OD has been intertwined. It is recommended that the introduction be rewritten to follow a chronology of thought. Minor comments include:

- There should be spacing between the square brackets and words. Eg. …global crises [1] NOT global crises[1]. This seems to appear throughout the write-up.
  - Line 71-71 It would be essential to state the most current prevalence of OD in SSA. What is the prevalence?
  - Line 77-78 Authors state OD is prevalent in parts of SSA? Authors should state or give examples of which countries of SSA.

3. The section on study area:

- Line 118 to 123 should be added as part of the introduction

4. The section on methodology: The methodology discussed is robust and reproducible. This gives credence to the data collected.
5. The section on Results and Discussion:

- Line 373 Separate bracket from “Leone”
- Lines 406 to 409: Authors provide reasons for the disparity in OD observed in SSA, Haiti and Indonesia. The reasons given are generic but written as though empirical. Authors should reword based on available evidence.
- Line 420 Check punctuations. Separate punctuations from words.
- Lines 430 to 436, Ghana is missing from the hotspot and coldspot classification. The authors measured access to water, and it will be interesting to highlight how this corresponds to the coldspot and hotspot classification of the OD region.
- Line 443-44 “Significant clusters were observed across nearly all regions of the region” should be reworded for clarity.
- Line 467 The GWR analysis predicted the influence of family size on OD. It would be interesting to triangulate family size, access to water and OD.
- Lines 467 to 480 Family size has a bearing on family income. Family income level determines creditworthiness and, by extension, access to targeted WASH loans for sanitation infrastructure. This could be a reason for the disparity in OD observed for different family sizes within the regions. Authors can consider this in the discussion.
  - Age bracket of head of household is too wide (11 to 35 years). Better inferences can be drawn if the age brackets are reduced further into more categories. For example, lower age brackets can influence inferences on household size, income, access to water and OD.

6. The section on Conclusion:

- The conclusions and recommendations seem to be an extension of the results and discussion. Authors must only highlight the novelty of the study, the key findings, and their implications for policy, practise, and future research.

7. Figures

- For Fig 1: Countries like Seychelles, Equatorial Guinea, Mauritius and Cape Verde have been duplicated
