# Supplementary material for: The prevalence, spatial distribution and geographic weighted regression of open defecation practice in sub-Saharan Africa using demographic and health survey (DHS) data
Source: PLoS One. 2025 Dec 17;20(12):e0336938. doi: 10.1371/journal.pone.0336938 (PMC12711022; doi:10.1371/journal.pone.0336938)
Supplement: Attachment — Submitted filename: Response to Reviewers.docx [file pone.0336938.s004.docx]

**Responses to the Editors and review’s comments**

**Dear PLOS ONE editorial team,**

Thank you for giving us the opportunity to submit a revised draft of the manuscript, and we would also like to thank you for your crucial comments on our paper (Manuscript ID: **PONE-D-** **25-12133R1**). We are very concerned and have combined all the suggested comments provided, which we believe strengthen our paper, and we hope this will render our paper eligible for consideration for publication in your reputed journal. We appreciate the time and effort that you and the reviewers dedicated to providing feedback on our manuscript and are grateful for the insightful comments and valuable improvements to our paper for publication.

The authors would like to inform you that we have addressed the comments and recommendations of the handling editor point by point. In addition, throughout our revision, we made our best corrections too. All changes made to the original version are highlighted using tracking changes and attached as **“Revised Manuscript with Track Changes”**. The unmarked copy of the manuscript is also attached as **“Manuscript”**. In addition, please see below a rebuttal letter that responds to each point raised by the handling editor, and this letter is also attached to the submission as **“Response to Reviewers”**.

**Response to editor’s comments**

**Comments from the handling editor:**

1.    I notice there are many figures, followed by 1-2 sentences explaining the figure, i.e., figure 5-16. Could you please combine 3-4 of them into two-three big figures, e.g., figure 5-8 to figure 5a-d (so 4 maps in 1 figure). And combine the paragraphs explaining those figures, so one paragraph does not consist only on 2 sentences.

**Author’s response:** Dear Editor, thank you very much for your recommendation. We have made the corrections accordingly to meet the journal requirements.

2.  Also, table 5 is not really necessarily in one specific table. You can put it under table 4.

**Author’s response:** Dear Editor, thank you very much for your recommendation. We have made the corrections accordingly to meet the journal requirements.

**Comments from Reviewer #1:**

1. Line 522	...increasing awareness of health risks related to open defecation [65].Such kind of families are... Authors should space the full stop from the word "such"

**Author’s response:** Thank you very much for your comment and suggestions. We have made corrections accordingly.
