# Supplementary material for: The prevalence, spatial distribution and geographic weighted regression of open defecation practice in sub-Saharan Africa using demographic and health survey (DHS) data
Source: PLoS One. 2025 Dec 17;20(12):e0336938. doi: 10.1371/journal.pone.0336938 (PMC12711022; doi:10.1371/journal.pone.0336938)
Supplement: Attachment — Submitted filename: Response_to_Reviewers_auresp_2.docx [file pone.0336938.s005.docx]

**Responses to the Editors and review’s comments**

**Dear PLOS ONE editorial team,**

Thank you for giving us the opportunity to submit a revised draft of the manuscript, and we would also like to thank you for your crucial comments on our paper (Manuscript ID: **PONE-D-25-12133**). We are very concerned and have combined all the suggested comments provided, which we believe strengthen our paper, and we hope this will render our paper eligible for consideration for publication in your reputed journal. We appreciate the time and effort that you and the reviewers dedicated to providing feedback on our manuscript and are grateful for the insightful comments and valuable improvements to our paper for publication.

The authors would like to inform you that we have addressed the comments and recommendations of the handling editor point by point. In addition, throughout our revision, we made our best corrections too. All changes made to the original version are highlighted using tracking changes and attached as **“Revised Manuscript with Track Changes”**. The unmarked copy of the manuscript is also attached as **“Manuscript”**. In addition, please see below a rebuttal letter that responds to each point raised by the handling editor, and this letter is also attached to the submission as **“Response to Reviewers”**.

**Response to editor’s comments**

**Comments from the handling editor:**

1.  Please ensure that your manuscript meets PLOS ONE's style requirements, including those for file naming. The PLOS ONE style templates can be found at <https://journals.plos.org/plosone/s/file?id=wjVg/PLOSOne_formatting_sample_main_body.pdf> and [https://journals.plos.org/plosone/s/file?id=ba62/PLOSOne_formatting_sample_title_authors_affiliations.pdf](https://track.editorialmanager.com/CL0/https:%2F%2Fjournals.plos.org%2Fplosone%2Fs%2Ffile%3Fid=ba62%2FPLOSOne_formatting_sample_title_authors_affiliations.pdf/1/010f01983bbebff0-43645a11-549f-44a7-a9b4-810f73eba355-000000/DTLr7emHGMIWI1tHuz1omOaYQBLZL3nwml5ZrIWtvgw=221)

**Author’s response:** Dear Editor, thank you very much for your recommendation. We have made the corrections accordingly to meet the journal requirements.

2. Please note that your Data Availability Statement is currently missing [the repository name and/or the DOI/accession number of each dataset OR a direct link to access each database]. If your manuscript is accepted for publication, you will be asked to provide these details on a very short timeline. We therefore suggest that you provide this information now, though we will not hold up the peer review process if you are unable. In your revision ensure you cite all your sources (including your own works), and quote or rephrase any duplicated text outside the methods section. Further consideration is dependent on these concerns being addressed.

**Author’s response:** Dear Editor, thank you very much for your recommendations. We have made the necessary corrections to meet the journal's requirements.

3.  [We note that Figures 1,  3, 4, 5, 6, 7, 8, 9, 10, 11, 12, 13, 14, 15 and 16 in your submission contain map/satellite images which may be copyrighted. All PLOS content is published under the Creative Commons Attribution License (CC BY 4.0), which means that the manuscript, images, and Supporting Information files will be freely available online, and any third party is permitted to access, download, copy, distribute, and use these materials in any way, even commercially, with proper attribution. For these reasons, we cannot publish previously copyrighted maps or satellite images created using proprietary data, such as Google software (Google Maps, Street View, and Earth). For more information, see our copyright guidelines: http://journals.plos.org/plosone/s/licenses-and-copyright.

We require you to either (1) present written permission from the copyright holder to publish these figures specifically under the CC BY 4.0 license, or (2) remove the figures from your submission:

a. You may seek permission from the original copyright holder of Figures 1,  3, 4, 5, 6, 7, 8, 9, 10, 11, 12, 13, 14, 15 and 16 to publish the content specifically under the CC BY 4.0 license.

We recommend that you contact the original copyright holder with the Content Permission Form (http://journals.plos.org/plosone/s/file?id=7c09/content-permission-form.pdf) and the following text:
“I request permission for the open-access journal PLOS ONE to publish XXX under the Creative Commons Attribution License (CCAL) CC BY 4.0 (http://creativecommons.org/licenses/by/4.0/). Please be aware that this license allows unrestricted use and distribution, even commercially, by third parties. Please reply and provide explicit written permission to publish XXX under a CC BY license and complete the attached form.”

Please upload the completed Content Permission Form or other proof of granted permissions as an "Other" file with your submission.

In the figure caption of the copyrighted figure, please include the following text: “Reprinted from [ref] under a CC BY license, with permission from [name of publisher], original copyright [original copyright year].”

b. If you are unable to obtain permission from the original copyright holder to publish these figures under the CC BY 4.0 license or if the copyright holder’s requirements are incompatible with the CC BY 4.0 license, please either i) remove the figure or ii) supply a replacement figure that complies with the CC BY 4.0 license. Please check copyright information on all replacement figures and update the figure caption with source information. If applicable, please specify in the figure caption text when a figure is similar but not identical to the original image and is therefore for illustrative purposes only.
The following resources for replacing copyrighted map figures may be helpful:

USGS National Map Viewer (public domain): http://viewer.nationalmap.gov/viewer/
The Gateway to Astronaut Photography of Earth (public domain): http://eol.jsc.nasa.gov/sseop/clickmap/
Maps at the CIA (public domain): https://www.cia.gov/library/publications/the-world-factbook/index.html and https://www.cia.gov/library/publications/cia-maps-publications/index.html
NASA Earth Observatory (public domain): http://earthobservatory.nasa.gov/
Landsat: http://landsat.visibleearth.nasa.gov/
USGS EROS (Earth Resources Observatory and Science (EROS) Center) (public domain): http://eros.usgs.gov/#
Natural Earth (public domain): http://www.naturalearthdata.com/](We%20note%20that%20Figures%201,%20 3,%204,%205,%206,%207,%208,%209,%2010,%2011,%2012,%2013,%2014,%2015%20and%2016%20in%20your%20submission%20contain%20map/satellite%20images%20which%20may%20be%20copyrighted.%20All%20PLOS%20content%20is%20published%20under%20the%20Creative%20Commons%20Attribution%20License%20(CC%20BY%204.0),%20which%20means%20that%20the%20manuscript,%20images,%20and%20Supporting%20Information%20files%20will%20be%20freely%20available%20online,%20and%20any%20third%20party%20is%20permitted%20to%20access,%20download,%20copy,%20distribute,%20and%20use%20these%20materials%20in%20any%20way,%20even%20commercially,%20with%20proper%20attribution.%20For%20these%20reasons,%20we%20cannot%20publish%20previously%20copyrighted%20maps%20or%20satellite%20images%20created%20using%20proprietary%20data,%20such%20as%20Google%20software%20(Google%20Maps,%20Street%20View,%20and%20Earth).%20For%20more%20information,%20see%20our%20copyright%20guidelines:%20http://journals.plos.org/plosone/s/licenses-and-copyright.We%20require%20you%20to%20either%20(1)%20present%20written%20permission%20from%20the%20copyright%20holder%20to%20publish%20these%20figures%20specifically%20under%20the%20CC%20BY%204.0%20license,%20or%20(2)%20remove%20the%20figures%20from%20your%20submission:a.%20You%20may%20seek%20permission%20from%20the%20original%20copyright%20holder%20of%20Figures%201,%20 3,%204,%205,%206,%207,%208,%209,%2010,%2011,%2012,%2013,%2014,%2015%20and%2016%20to%20publish%20the%20content%20specifically%20under%20the%20CC%20BY%204.0%20license.We%20recommend%20that%20you%20contact%20the%20original%20copyright%20holder%20with%20the%20Content%20Permission%20Form%20(http://journals.plos.org/plosone/s/file?id=7c09/content-permission-form.pdf)%20and%20the%20following%20text:)

**Author’s response:** Dear Editor, thank you very much for your recommendation. We have made the corrections accordingly to meet the journal requirements.

**This is Global Administrative Areas Database (GADM) license**

The data are freely available for academic use and other non-commercial use. Redistribution or commercial use is not allowed without prior permission. Using the data to create maps for publishing of academic research articles is allowed. Thus you can use the maps you made with GADM data for figures in articles published by PLoS, Springer Nature, Elsevier, MDPI, etc. You are allowed (but not required) to publish these articles (and the maps they contain) under an open license such as CC-BY as is the case with PLoS journals and may be the case with other open access articles. Data for the following countries is covered by a a different license Austria: Creative Commons Attribution-Share Alike 2.0 (source: Government of Ausria)

You can find this information through this link: <https://gadm.org/license.html>

In addition previous study also used maps from GADM here it is the link: <https://journals.plos.org/plosone/article?id=10.1371/journal.pone.0318189>

4. If the reviewer comments include a recommendation to cite specific previously published works, please review and evaluate these publications to determine whether they are relevant and should be cited. There is no requirement to cite these works unless the editor has indicated otherwise.

**Author’s response:** Dear Editor, thank you very much for your recommendation. We have made the corrections accordingly to meet the journal requirements.

**Comments from Reviewer #1:**

1. The study is technically sound, with data largely supporting conclusions. However, clarify causality limitations due to cross-sectional design and address counterintuitive findings

**Author’s response:** Thank you very much for your comment and suggestions. We have made corrections accordingly.

2. Generally acceptable with minor clarifications needed. Spatial analyses are robust, but provide:- Timeframe of DHS data per country

**Author’s response:** Thank you very much for your comment and suggestions. We have made corrections accordingly.

3. Multicollinearity assessment in GWR, Criteria for cluster/household inclusion.

**Author’s response:** Thank you very much for your comment and suggestions. We have made corrections accordingly. We have included statement in the “Spatial regression analysis” section of the manuscript.

Clusters and households were included in the analysis if they had complete data on the outcome variable (open defecation status) and all selected explanatory variables. The analysis was conducted using the Household Recode (HR) files from the DHS datasets. Clusters with missing spatial coordinates or implausible values were excluded to maintain spatial accuracy.

The sanitation variable was extracted from the HR file using the DHS variable code hv205, which indicates the type of toilet facility used by the household. This variable was recoded into a binary outcome: households were classified as practicing open defecation (Yes) if hv205 = no facility/bush/field, and No otherwise (i.e., any other improved or unimproved facility

4. Data Repository Compliance. Data availability statement is appropriate, citing DHS program access. Ensure supplementary country-level statistics are included.

**Author’s response:** Thank you very much for your comment and suggestions. We have made corrections accordingly. We have included country-level statistics as S1 File.

5. Presentation & Language Generally intelligible but requires: - Minor English editing for conciseness Higher-resolution maps with clear legends/scales.

**Author’s response:** Thank you very much for your comment and suggestions. We have made corrections accordingly.

6. Specify temporal range of DHS datasets used.

**Author’s response:** Thank you very much for your comment and suggestions. We have made corrections accordingly

7. Detail handling of missing data and variable coding.

**Author’s response:** Thank you very much for your comment and suggestions. We have made corrections accordingly in data monument **and analysis** section of the manuscript.

8. Please clarify the time frame of the DHS data included for each country. Specify inclusion/exclusion criteria for clusters and households, and provide more detail on how variables were selected and handled in the GWR, including how you addressed multicollinearity

**Author’s response:** Thank you very much for your comment and suggestions. We have made corrections accordingly

9. The conclusion that larger households are negatively associated with open defecation may seem counterintuitive and could benefit from referencing cultural/family dynamics or sanitation sharing behavior.

**Author’s response:** Thank you very much for your comment and suggestions. We have made corrections accordingly

10. Statements like “richest households negatively associated” need caution:-correlation ≠ causation, and the pathways should be clarified.

**Author’s response:** Thank you very much for your comment and suggestions. We have made corrections accordingly. We change the phrase from **correlation to negative association.**

11. Explain mechanisms driving East/West African hotspots beyond statistical identification.

**Author’s response:** Thank you very much for your comment and suggestions. We have made corrections accordingly.

12. The spatial analyses are robust, but the discussion would benefit from deeper interpretation of the drivers behind identified hotspots and coldspots, and more explicit policy implications for regions with high prevalence

**Author’s response:** Thank you very much for your comment and suggestions. We have made corrections accordingly is discussion section of manuscript.

13. Emphasize cross-sectional limitations regarding causal inference in discussion. Discuss potential unmeasured confounders

**Author’s response:** Thank you very much for your comment and suggestions. We have made corrections accordingly is discussion section of manuscript..

14. The finding that both urban and rural residency are negatively associated with open defecation is counterintuitive. Please clarify this result and discuss possible explanations or limitations in the data or analytic approach.

**Author’s response:** Thank you very much for your comment and suggestions. We have made corrections accordingly.

15. Tables/Figures:- Ensure that all figures/maps are high-resolution and include legends and scales for clarity.

**Author’s response:** Thank you very much for your comment and suggestions. We have made corrections accordingly and all figures/maps complies with journal guideline.

16. Ethics and Data Statements:- Assessment:-Fully compliant. The ethics and data availability statements are appropriate and transparent, but consider explicitly listing the countries and IRBs where possible IRBs. Clearly stated that this is a secondary analysis of DHS data, which had ethical clearance and informed consent in all countries.

**Author’s response:** Thank you very much for your comment and suggestions. We have made corrections accordingly.

17. Language:- The manuscript is generally clear but would benefit from minor English language editing for conciseness and flow.

**Author’s response:** Thank you very much for your comment and suggestions. We have made some corrections accordingly.

18. Suggestions Include a limitations subsection addressing:- Regional data gaps (e.g., unstable regions not covered) Temporal variations in DHS collection. Expand policy recommendations targeting hotspot interventions.

**Author’s response:** Thank you very much for your comment and suggestions. We have made some corrections accordingly. We have included a statement in the recommendation and limitation section of the manuscript.

**Comments from Reviewer #2:**

1. The conclusion is a repeat of the methodology and the last sentence of the background. The conclusion should highlight the key finding(s) and their implication on policy and future research. What makes this study novel?

**Author’s response:** Thank you very much for your comment and suggestions. We have made some corrections accordingly. We included a statement In conclusion section of the manuscript.

2. The study asserts 23.24% of open defecation practices in Sub-Saharan Africa. How does this compare to the value obtained by the JMP? This should be highlighted briefly.

**Author’s response:** Thank you very much for your comment and suggestions. We have made some corrections accordingly. We included a statement In conclusion section of the manuscript.

3. There should be spacing between the square brackets and words. Eg. …global crises [1] NOT global crises[1]. This seems to appear throughout the write-up.

**Author’s response:** Thank you very much for your comment and suggestions. We have made some corrections accordingly.

4. Line 71-71 It would be essential to state the most current prevalence of OD in SSA. What is the prevalence?

**Author’s response:** Thank you very much for your comment and suggestions. We have made corrections accordingly in line number 95-96.

5. Line 77-78 Authors state OD is prevalent in parts of SSA? Authors should state or give examples of which countries of SSA.

**Author’s response:** Thank you very much for your comment and suggestions. We have made corrections accordingly in line number 78-81

6. The section on study area: Line 118 to 123 should be added as part of the introduction

**Author’s response:** Thank you very much for your comment and suggestions. We have made corrections accordingly.

7. Line 373 Separate bracket from “Leone”

**Author’s response:** Thank you very much for your comment and suggestions. We have made corrections accordingly.

8. Lines 406 to 409: Authors provide reasons for the disparity in OD observed in SSA, Haiti and Indonesia. The reasons given are generic but written as though empirical. Authors should reword based on available evidence.

**Author’s response:** Thank you very much for your comment and suggestions. We have made corrections accordingly.

9. Line 420 Check punctuations. Separate punctuations from words.

**Author’s response:** Thank you very much for your comment and suggestions. We have made corrections accordingly.

10. Lines 430 to 436, Ghana is missing from the hotspot and coldspot classification. The authors measured access to water, and it will be interesting to highlight how this corresponds to the coldspot and hotspot classification of the OD region.

**Author’s response:** Thank you very much for your comment and suggestions. We would like to clarify that the section in question refers to the **Cluster and Outlier Analysis** of open defecation practices in sub-Saharan Africa, which is presented in **Figure 6**. This analysis is distinct from the **hotspot and coldspot classification** conducted using the Getis-Ord Gi* statistic. According to Figure 6, Ghana falls under **high-low** and **low-high** outlier categories. However, our primary focus is on the **high-high** and **low-low** clusters, as these indicate areas with consistent spatial patterns of high or low open defecation prevalence. This approach aligns with the analytical focus adopted in several previous studies using similar methods. In addition, several other countries are also found in the **high-low** and **low-high** outlier categories Ghana is not the only country.

11. Line 443-44 “Significant clusters were observed across nearly all regions of the region” should be reworded for clarity.

**Author’s response:** Thank you very much for your comment and suggestions. We have made corrections accordingly.

12. Line 467 The GWR analysis predicted the influence of family size on OD. It would be interesting to triangulate family size, access to water and OD.

**Author’s response:** Thank you very much for your comment and suggestions. We have made corrections accordingly. We have included a statement about it in the discussion section of the manuscript from line 540-545.

13. Lines 467 to 480 Family size has a bearing on family income. Family income level determines creditworthiness and, by extension, access to targeted WASH loans for sanitation infrastructure. This could be a reason for the disparity in OD observed for different family sizes within the regions. Authors can consider this in the discussion

**Author’s response:** Thank you very much for your comment and suggestions. We have made corrections accordingly. We have included a statement about it in the discussion section of the manuscript from line 528-533.

14. Age bracket of head of household is too wide (11 to 35 years). Better inferences can be drawn if the age brackets are reduced further into more categories. For example, lower age brackets can influence inferences on household size, income, access to water and OD.

**Author’s response:** Thank you very much for your comment and suggestions.

However, our classification was based on previous studies. We selected this approach to categorize variables in a way that aligns with established literature and facilitates meaningful analysis. For example check this research: <https://www.frontiersin.org/journals/public-health/articles/10.3389/fpubh.2024.1420077/full>

15. The conclusions and recommendations seem to be an extension of the results and discussion. Authors must only highlight the novelty of the study, the key findings, and their implications for policy, practise, and future research.

**Author’s response:** Thank you very much for your comment and suggestions. We have made corrections accordingly. We have included a statement about it in the Conclusion and recommendations section of the manuscript.

16. For Fig 1: Countries like Seychelles, Equatorial Guinea, Mauritius and Cape Verde have been duplicated

**Author’s response:** Thank you very much for your comment and suggestions. The countries such as Seychelles, Equatorial Guinea, Mauritius, and Cape Verde are island nations, and some consist of multiple islands. As a result, they may appear more than once in Figure 1 due to their geographic distribution.
